# Lung mechanics in type L CoVID-19 pneumonia: a pseudo-normal ARDS

**DOI:** 10.1186/s41231-020-00076-9

**Published:** 2020-12-21

**Authors:** Lorenzo Viola, Emanuele Russo, Marco Benni, Emiliano Gamberini, Alessandro Circelli, Luca Bissoni, Domenico Pietro Santonastaso, Giovanni Scognamiglio, Giuliano Bolondi, Luca Mezzatesta, Vanni Agnoletti

**Affiliations:** 1 U.O. Anestesia e Rianimazione, Ospedale “M. Bufalini” Hospital, 286, Viale Ghirotti, Cesena, Italy; 2 University of Messina, Messina, Italy

**Keywords:** CoVID-19 pneumonia, transpulmonary pressure, prone positioning, mechanical power.

## Abstract

**Background:**

This study was conceived to provide systematic data about lung mechanics during early phases of CoVID-19 pneumonia, as long as to explore its variations during prone positioning.

**Methods:**

We enrolled four patients hospitalized in the Intensive Care Unit of “M. Bufalini” hospital, Cesena (Italy); after the positioning of an esophageal balloon, we measured mechanical power, respiratory system and transpulmonary parameters and arterial blood gases every 6 hours, just before decubitus change and 1 hour after prono-supination.

**Results:**

Both respiratory system and transpulmonary compliance and driving pressure confirmed the pseudo-normal respiratory mechanics of early CoVID-19 pneumonia (respectively, C_RS_ 40.8 ml/cmH_2_O and DP_RS_ 9.7 cmH_2_O; C_L_ 53.1 ml/cmH_2_O and DP_L_ 7.9 cmH_2_O). Interestingly, prone positioning involved a worsening in respiratory mechanical properties throughout time (C_RS,SUP_ 56.3 ml/cmH_2_O and C_RS,PR_ 41.5 ml/cmH_2_O – P 0.37; C_L,SUP_ 80.8 ml/cmH_2_O and C_L,PR_ 53.2 ml/cmH_2_O – P 0.23).

**Conclusions:**

Despite the severe ARDS pattern, respiratory system and lung mechanical properties during CoVID-19 pneumonia are pseudo-normal and tend to worsen during pronation.

**Trial registration:**

Restrospectively registered.

## Background

Since its outbreak, in January, 2020, it has been clear that CoVID-19 pneumonia is atypical. Despite a full concordance to Berlin criteria for Acute Respiratory Distress Syndrome (ARDS), respiratory system mechanics is preserved [1]. Mechanical ventilation and muscular paralysis are recommended in worsening respiratory insufficiency [2]; in a substantial number of cases, prone positioning significantly improves oxygenation.

Little is known about isolated lung behavior in CoVID-19 pneumonia. Hence, the aim of this study is to analyze lung mechanical properties in the first hours after the beginning of mechanical ventilation and in prone and supine position.

## Methods

A retrospective observational study was performed at Maurizio Bufalini hospital (Cesena, Italy). Patients hospitalized in the Intensive Care Unit (ICU) from 03/23/2020 to 04/10/2020 were enrolled. The inclusion criteria were: age > 18 years, need of mechanical ventilation, need of muscular paralysis and < 48 hours of tracheal intubation.

After admission in ICU, a naso-gastric tube with an esophageal balloon (Nutrivent® - SEDA S.p.A., Mirandola, Italy) was positioned; the correct positioning and insufflation volume were tested with the occlusion method and measures were recorded with a multiparametric monitor connected to esophageal balloon and ventilator circuit (Optivent® – SEDA S.p.A., Mirandola, Italy).

Protective ventilation, defined as tidal volume (V_t_) of 5–7 ml/kg predicted body weight (PBW), was used. Respiratory rate (RR) was set to tolerate mild hypercarbia (p_a_CO_2_ <60 mmHg) and/or pH > 7.25.

Measures were performed at admission, then every six hours or just before placing patients in prone or supine position and one hour after the change of decubitus. An arterial blood gas sample was collected along with every evaluation. We stopped measuring when muscular paralysis was suspended. Ventilator settings were recorded; static parameters were obtained through a 3 seconds inspiratory and expiratory hold. Airway (P_AW_) and esophageal (P_ES_) pressure values were recorded and the latter was used to calculate transpulmonary pressure (P_L_), as the result of the real-time subtraction of P_ES_ to P_AW_. Subsequently, compliance (C_RS_, C_L_), driving pressure (DP_RS_, DP_L_) and mechanical power (MP_RS_, MP_L_) related both to respiratory system and lung were calculated [3].

## Results

We report data of four consecutive patients who fulfilled the inclusion criteria; two more patients were enrolled and excluded from the analysis – one died and the other was suspended myoresolution after enrollment. In all patients, chest computed tomography (CT) showed interstitial pneumonia without loss of parenchymal aeration; patients were put on mechanical ventilation within 24 hours of hospital admission. In Table 1 are summarized the main clinical and ventilatory features for every patient.

**Table 1:** Clinical features, ventilator settings and mechanical measurements of individual patients, in supine and prone position. BMI: Body Mass Index; I:E: inspiratory-to-expiratory ratio; PEEP: Positive End-Expiratory Pressure; P_plat_: plateau pressure; PEEP_tot_: total PEEP; DP_RS_: Respiratory System Driving Pressure; C_RS_: Respiratory System Compliance; MP_RS_: Respiratory System Mechanical Power; P_L,end insp_: Transpulmonary Pressure at end inspiration; P_L,end exp_: Transpulmonary Pressure at end expiration; DP_L_: Transpulmonary Driving Pressure; C_L_: Transpulmonary Compliance; MP_L_: Transpulmonary Mechanical Power; p_a_O_2_: oxygen arterial partial pressure; F_I_O_2_: inspired fraction of oxygen; p_a_CO_2_: carbon-dioxide arterial partial pressure; A-aO_2_ gradient: alveolar-to-arterial oxygen gradient; EtCO_2_/p_a_CO_2_ ratio: end-tidal CO_2_ to p_a_CO_2_ ratio

	*Patient 1*		*Patient 2*		*Patient 3*			*Patient 4*		
	Supine Median (IQR)	Prone Median (IQR)	Supine Median (IQR)	Prone Median (IQR)		Supine Median (IQR)	Prone Median (IQR)		Supine Median (IQR)	Prone Median (IQR)
*Clinical features*										
Age	67		43		75			65		
Sex	M		M		F			M		
BMI (kg/m^2^)	24.5		28,4		31,3			24,8		
Comorbidities	No		No		BPCO			No		
*Ventilator settings*										
Tidal volume (ml/PBW)	5.9 (0)	6.2 (0.04)	6.1 (0.3)	5.9 (0)		7.4 (0.1)	7,4 (0)		5.6 (0.2)	5.6 (0)
Respiratory rate (bpm)	22 (4.5)	19.5 (2.5)	20 (0.3)	20 (0)		20 (0)	20 (0)		28 (5)	25 (8)
I:E (sec)	0.51 (0.2)	0.48 (0.11)	0.56 (0.11)	0.64 (0.13)		0.5 (0)	0.5 (0)		0.43 (0.07)	0.53 (0.07)
PEEP (cmH_2_O)	12 (3)	12 (0)	10 (1)	11 (1)		14 (0)	14 (0)		10 (2)	12 (0)
*Respiratory system mechanics*										
P_plat_ (cmH_2_O)	19 (3)	20 (2.1)	21.8 (2.3)	21.5 (1.5)		23.6 (0.6)	24.7 (0.3)		23 (2.3)	27 (3)
PEEP_tot_ (cmH_2_O)	12.4 (2.5)	12.7 (0.4)	10.9 (0.9)	11.7 (1.3)		14.9 (0.2)	14.9 (0.1)		11 (3.7)	13 (2)
DP_RS_ (cmH_2_O)	7 (0.7)	7.8 (1.4)	10.9 (1.4)	9.8 (0.2)		8.7 (0.7)	9.8 (0.3)		12 (1.8)	13.8 (1)
C_RS_ (ml/cmH_2_O)	60.1 (6.3)	57.2 (17.7)	38.3 (3.6)	39.8 (0.8)		44.6 (4.2)	40 (1)		35.4 (4.7)	33.1 (2.3)
MP_RS_ (J/min)	16.9 (4.8)	16.4 (2)	15.7 (3.3)	15.8 (2.3)		17.9 (0.1)	18.2 (0)		25.6 (7.2)	27.6 (8.8)
*Lung mechanics*										
P_L_, end insp (cmH_2_O)	8.2 (3.2)	7.9 (3)	13.5 (2.2)	16 (1.9)		11.4 (2.6)	15.3 (0.2)		14.3 (1.5)	19.6 (7.6)
P_L_, end exp (cmH_2_O)	4.4 (2.2)	3 (1.5)	4.4 (2)	7.6 (1.6)		6.1 (2.1)	7.7 (0.5)		5.4 (1.7)	7.9 (5.8)
DP_L_ (cmH_2_O)	4.4 (2.4)	5.2 (2.1)	9.2 (1.5)	8.4 (0.3)		5.4 (0.6)	7.6 (0.6)		8.8 (2.3)	11.5 (1.4)
C_L_ (ml/cmH_2_O)	100.4 (54.5)	81.5 (23.3)	45.2 (7.4)	46.5 (1.7)		72.8 (8.4)	51.6 (4.1)		47.7 (9.6)	39.3 (4.6)
MP_L_ (J/min)	11.8 (2.5)	12.4 (1.2)	12.2 (1.6)	11.6 (0.9)		12.6 (0)	13.6 (0.2)		16.6 (4)	20.1 (6.8)
*Blood gas analysis*										
P_a_O_2_ (mmHg)	90.6 (13.3)	84.6 (53.6)	75.3 (16.8)	82.3 (12.4)		141.8 (54)	164.6 (16.9)		64.5 (17.8)	75.9 (16.9)
F_I_O_2_ (%)	80 (22.5)	75 (17.5)	50 (2.5)	50 (0)		77.5 (22.5)	70 ()		80 (10)	80 (5)
P_a_CO_2_ (mmHg)	68 (12.1)	58.1 (10.1)	50.4 (1.5)	40.6 (0.2)		53 (5.6)	45.3 (5.3)		52.1 (7.7)	53 (10.3)
pH	7.23 (0.05)	7.28 (0.06)	7.38 (0.03)	7.41 (0.01)		7.35 (0.04)	7.37 (0)		7.22 (0.05)	7.24 (0.06)
A-aO_2_ gradient (mmHg)	386.6 (153.1)	325.7 (47.1)	219.5 (5.3)	223.5 (12.2)		344.6 (113.4)	277.9 (35.2)		372.5 (71)	433.9 (82.3)
EtCO_2_/p_a_CO_2_ ratio	0.51 (0.09)	0.68 (0.25)	0.84 (0.03)	0.84 (0)		0.64 (0.05)	0.74 (0.19)		0.58 (0.06)	0.57 (0.01)

The median time of observation was 54.5 hours. Patients underwent 1.5 median cycles of prono-supination, for a median pronation time of 17 (IQR 7) hours per cycle. Median V_t_ was 5.9 (IQR 0.5) ml/kg PBW and median RR was 20 (IQR 4.8) breaths per minute; MP_RS_ was 17.9 (IQR 7.1) J/min, while MP_L_ was 13.1 (2.8) J/min. Median C_RS_ and DP_RS_ were, respectively, 40.8 (IQR 19.4) ml/cmH_2_O and 9.7 (IQR 4) cmH_2_O. The same parameters, calculated using the transpulmonary pressure, led to a median C_L_ of 53.1 (IQR 35) ml/cmH_2_O and a median DP_L_ of 7.9 (IQR 3.9) cmH_2_O.

The values reported in Table 1 referring to prone and supine position represent the median of all the measurements done during the entire duration of the decubitus, independently of the time passed from decubitus change and the number of pronation cycles performed. No statistically significant variation was observed in respiratory system (C_RS,SUP_ 40.9 (IQR 21.6) ml/cmH_2_O; C_RS,PR_ 40.6 (IQR 14.1) ml/cmH_2_O – P 0.93) and lung (C_L,SUP_ 55.6 (IQR 35.8) ml/cmH_2_O; C_L,PR_ 48.9 (IQR 26.2) ml/cmH_2_O – P 0.7) mechanics during prone positioning.

## Discussion

CoVID-19 pneumonia is peculiar: despite a severe hypoxemia, respiratory system mechanics is pseudo-normal [1]. Gattinoni et al.. described a biphasic trend of the CoVID-19 pneumonia: in the initial phase – type L pneumonia – elastance is low, as well as recruitability, ventilation/perfusion ratio (V/Q ratio) and lung weight on CT scan. Conversely, in the second phase – type H pneumonia (20–30% of cases) – elastance, recruitability and lung weight are high and right-to-left shunt predominates [4, 5], thus framing in a classical form of ARDS. However, data regarding isolated lung mechanical properties in type L pneumonia are partial and disorganized.

We present preliminary data of a series of patients affected by type L pneumonia. Through the systematic evaluation of transpulmonary pressure, our findings seem to confirm the pseudo-normality of lung mechanics during the first days of mechanical ventilation and in different clinical settings. Even if lungs were severely damaged, the transpulmonary pressures remained below the thresholds commonly referred to as harmful [6], confirming a preserved lung aeration.

Another proof of the pseudo-normality of the respiratory system comes from the calculation of mechanical power [3]. Serpa Neto and coworkers found that risk for ventilation-induced lung injury (VILI) starts to increase from a value above 17 J/min [7]. Despite high ventilatory requests for maintaining acceptable p_a_CO_2_ and pH, in our series MP_RS_ remained at a borderline value of 17.9 J/min. In an experimental study, Cressoni et al. found that VILI occurs with a MP_L_ above 12 J/min [8]; our data show a median MP_L_ of 13.1 J/min, that is slightly above the harmful value. Therefore, while standard protective ventilation is unlikely to lead to VILI, we cannot clearly define whether the ventilatory demands in type L pneumonia are injurious to the lung.

**Fig. 1:**
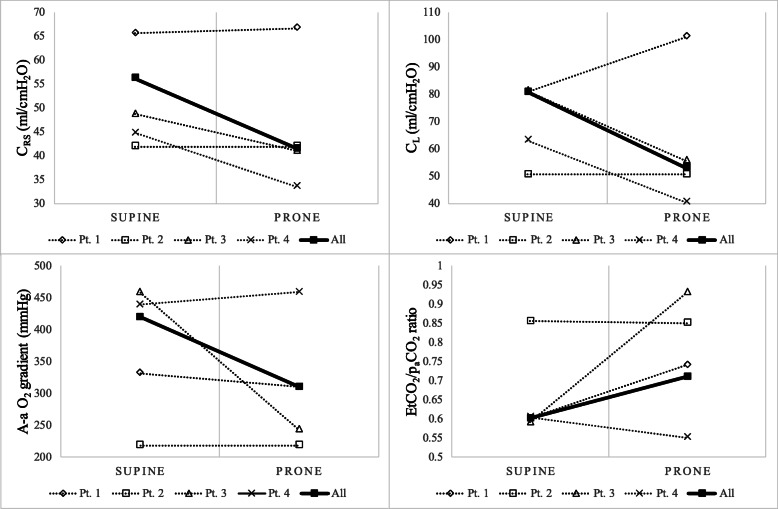
Mechanical and Ventilation/Perfusion ratio variations in supine and prone positioning, just before decubitus change. C_RS_: Respiratory System Compliance; C_L_: Transpulmonary Compliance; A-aO_2_ gradient: alveolar-to-arterial oxygen gradient; EtCO_2_/p_a_CO_2_ ratio: end-tidal CO_2_ to p_a_CO_2_ ratio

Type L pneumonia in characterized by a profound hypoxemia, in most cases dramatically responsive to pronation. Prone positioning involves a redistribution of transpulmonary pressure throughout the lung and an inflation improvement in well perfused dorsal areas, leading to an amelioration of ventilation/perfusion ratio (V/Q ratio) [9]. We evaluated the V/Q ratio through alveolar-to-arterial oxygen gradient (A-aO_2_ gradient) and end-tidal CO_2_/p_a_CO_2_ ratio (EtCO_2_/p_a_CO_2_ ratio) [5, 10] both in prone and supine positioning, just before the decubitus change. As expected, pronation entails a reduction of V/Q mismatch (A-aO_2_ gradient_SUP_ 419 mmHg; A-aO_2_ gradient_PR_ 310 mmHg – P 0.29; EtCO_2_/p_a_CO_2_ ratio_SUP_ 0.6; EtCO_2_/p_a_CO_2_ ratio_PR_ 0.71 – P 0.63). Interesting data come from the analysis of the last measurements done before the variation of the positioning, which account for the extreme consequences of prone vs. supine positioning; indeed, whereas median lung and respiratory compliance in prone and supine position does not substantially differ, C_RS_and C_L_worsen throughout pronation time (C_RS,END SUP_56.3 ml/cmH_2_O vs. C_RS,END PR_41.5 ml/cmH_2_O – P 0.37; C_L,END SUP_80.8 ml/cmH_2_O vs. C_L,END PR_53.2 ml/cmH_2_O – P 0.23; see Fig. 1).

In previous studies on primary ARDS, prone positioning was associated with a decrease of chest wall compliance (C_CW_), but did not substantially affect C_RS_ nor C_L_ [9]. In a review published in 2018, Guérin and Mezidi investigated the effect of patient positioning on respiratory mechanics in critical patients under mechanical ventilation. Prone position resulted in an increase of C_RS_ in three of the eight trials analyzed; transpulmonary pressure was measured only in five studies: in two of them, C_L_ increased; in the remaining three studies, C_L_ was unchanged [11]. There are at least two differences between those studies and our work: first of all, time in prone positioning was considerably lower - from few minutes to 2 hours vs. a median of 17 hours; second, all the studies included patients with “classical” ARDS, whose radiological, mechanical and clinical features differ from CoVID-19 pneumonia.

The effect of prone positioning is currently explained by the “sponge model” [12]. According to this model, the inflammatory alveolar edema that characterizes ARDS exerts a hydrostatic pressure that compresses the dependent dorsal lung parenchyma and squeezes out the gas content; prone positioning causes a reversal of this condition: edema tends to compress the ventral lung fields, promoting the reopening of the dorsal areas. This phenomenon accounts for the dubious impact of prone positioning on C_RS_ and C_L_. According to radiological data, in early CoVID-19 pneumonia the edematous component is less represented and it may be assumed that, as the sponge model fails, the pathophysiological basis of the pronation effect will change. The reasons why C_L_ and consequently C_RS_ decrease during prone positioning in CoVID-19 patients have yet to be investigated: a hypothesis could be that pronation carries out a re-organization of incipient lung edema with a resulting increase in lung elastance. CT studies, as well as the application of electric impedance tomography, may help to verify this theory.

The clinical consequence of this pseudo-normal behavior of respiratory mechanics during prone position should also be clarified. As expected, we registered a worsening trend both in respiratory system and lung mechanical power (MP_RS,SUP_ 17.4 J/min vs. MP_RS,PR_ 18 J/min – P 0.53 and MP_L,SUP_ 12.5 J/min vs. MP_L,PR_ 14.1 J/min – P 0.29), indicating a greater risk of VILI induction with prolonged prone positioning.

Given the small number of enrolled patients this study is meant to be a pilot study. To reach statistical significance a sample size of 14 subject is needed (Power analysis with alpha 0.05 and Power 80%). Our study lacks the statistical power to verify our findings; as well, we can only hypothesize the reason why we registered those changes in respiratory mechanics. Thus, future studies should aim to the statistical validation of our data and, secondly, to their explanation.

## Conclusions

Our data underline the differences between classical ARDS and type L pneumonia, characterized by a pseudo-normal lung mechanics that deteriorates during prone positioning. This paper would be a “call for research” in this emerging topic; we firmly believe that understanding the unusual pathophysiology of severe respiratory failure in SARS-CoV-2 infected patients is the key to effectively treating these critically ill patients.

## Data Availability

The datasets used and/or analysed during the current study are available from the corresponding author on reasonable request.

## Abbreviations

A-aO_2_ gradient: Alveolar-to-arterial Oxygen gradient
ARDS: Acute Respiratory Distress Syndrome
C_CW_: Chest Wall Compliance
C_RS_: Respiratory System Compliance
C_RS,SUP_: Respiratory System Compliance, Supine
C_RS,PR_: Respiratory System Compliance, Prone
C_L_: Transpulmonary Compliance
C_L,SUP_: Transpulmonary Compliance, Supine
C_L,PR_: Transpulmonary Compliance, Prone
CoVID-19: Coronavirus Infectious Disease-19
CT: Computed Tomography
DP_RS_: Respiratory System Driving Pressure
DP_L_: Transpulmonary Driving Pressure
EtCO_2_/p_a_CO_2_ ratio: end-tidal CO_2_/p_a_CO_2_ ratio
ICU: Intensive Care Unit
MP_RS_: Respiratory System Mechanical Power
MP_L_: Transpulmonary Mechanical Power
p_a_CO_2_: carbon dioxide arterial partial pressure
P_AW_: Airway Pressure
PBW: Predicted Body Weight
P_ES_: Esophageal Pressure
P_L_: Transpulmonary pressure
RR: Respiratory Rate
V/Q ratio: Ventilation/Perfusion ratio
VILI: Ventilation-Induced Lung Injury
V_t_: Tidal Volume
